# Job Displacement and First Birth Over the Business Cycle

**DOI:** 10.1007/s13524-017-0580-4

**Published:** 2017-06-05

**Authors:** Barbara Hofmann, Michaela Kreyenfeld, Arne Uhlendorff

**Affiliations:** 1 Institute for Employment Research (IAB), Regensburger Straße 104, 90478 Nuremberg, Germany; 2 Hertie School of Governance, Berlin, Germany; 3 Max Planck Institute for Demographic Research, Konrad-Zuse-Str. 1, 18057 Rostock, Germany; 4 CREST, CNRS, Université Paris Saclay, 15 Boulevard Gabriel Peri, 92245 Malakoff Cedex, France

**Keywords:** Business cycle, Fertility, Job loss, Mass layoffs, Unemployment

## Abstract

In this article, we investigate the impact of job displacement on women’s first-birth rates as well as the variation in this effect over the business cycle. We use mass layoffs to estimate the causal effects of involuntary job loss on fertility in the short and medium term, up to five years after displacement. Our analysis is based on rich administrative data from Germany, with an observation period spanning more than 20 years. We apply inverse probability weighting (IPW) to flexibly control for the observed differences between women who were and were not displaced. To account for the differences in the composition of the women who were displaced in a downturn and the women who were displaced in an upswing, we use a double weighting estimator. Results show that the extent to which job displacement has adverse effects on fertility depends on the business cycle. The first-birth rates were much lower for women who were displaced in an economic downturn than for those who lost a job in an economic upturn. This result cannot be explained by changes in the observed characteristics of the displaced women over the business cycle.

**Electronic supplementary material:**

The online version of this article (doi:10.1007/s13524-017-0580-4) contains supplementary material, which is available to authorized users.

## Introduction

Generally, adverse economic conditions and increasing unemployment rates are expected to lead to a postponement of birth decisions and thus to a decline in period fertility rates at the aggregate level (Adserà 2004; Currie and Schwandt 2014; Goldstein et al. 2013; Lanzieri 2013; Luci and Thévenon 2009; Neels et al. 2013; Sobotka et al. 2011). However, the micro-level evidence on this relationship is ambiguous, often showing little or no association between individual unemployment and birth rates (Andersson 2000; Gutiérrez-Domènech 2008; Kravdal 2002; Kreyenfeld and Andersson 2014; Matysiak and Vignoli 2013; Özcan et al. 2010; Pailhé and Solaz 2012; Schmitt 2012; Vignoli et al. 2012). Potential reasons for the lack of clear micro-level evidence are the endogeneity of unemployment and the fact that employment and fertility outcomes can be jointly determined, thus implying that the observed and the unobserved characteristics of women who become unemployed differ systematically from those of women who do not become unemployed. Not taking these differences into account can lead to biased estimates (Moffitt 2005).

This article is a response to the call for more studies that model the causal impact of adverse economic conditions on fertility behavior. We estimate the effect of being displaced from a job on a woman’s decision about whether to have a first child. The main contribution of this study is to provide micro-level evidence on the question of whether experiencing job displacement during a recession affects a woman’s fertility differently than losing a job during an economic upswing. In our investigation, we borrow from labor economics research comparing the causal impact of labor market programs on employment behavior across time (Heinrich and Mueser 2014; Lechner and Wunsch 2009; Rinne et al. 2013). In addition, we draw on the economic literature that uses mass layoffs and plant closures to disentangle voluntary and involuntary job loss (see, e.g., Jacobson et al. 1993; Song and von Wachter 2014). Recently, this strategy has also received attention in fertility studies (Del Bono et al. 2012, 2015; Huttunen and Kellokumpu 2015). We use an approach similar to those applied in these earlier studies: that is, we assume that a mass layoff or a plant closure represents an exogenous shock that can generate economic uncertainty in a woman’s life course. To flexibly control for differences in the observed characteristics of the women who lost a job due to a mass layoff or a plant closure and of the women who were not affected by such an event, we apply inverse probability weighting (IPW) estimations (see Gangl 2010; Imbens and Wooldridge 2009; Morgan and Winship 2015 for a discussion of IPW estimators).

An involuntary job loss can have an impact on the employment and fertility decisions of a woman through different channels. On the one hand, a job loss can lead to a loss of income. All else being equal, this income loss may lead to a postponement of childbearing. For example, Lindo (2010) provided evidence that the income shock resulting from the job displacement of a woman’s husband had a negative effect on her fertility. On the other hand, an unemployment episode following a job displacement may reduce the opportunity costs of raising children and thus increase a displaced woman’s fertility (Becker 1965, 1993; Butz and Ward 1979).

This basic economic approach does not factor in women’s long-term employment plans or the option to outsource childcare. According to a complementary, sociologically oriented hypothesis, parenthood can be interpreted as a rational choice that reduces biographical uncertainties (Friedman et al. 1994). An unemployed woman who perceives that she has few opportunities to “succeed in the mainstream economy” (McDonald 2000:10) may seize on motherhood as a predictable and rewarding strategy for structuring her otherwise uncertain life course. Like the basic economic model, this framework suggests that women will be more likely to give birth during periods of unemployment. However, researchers have also posited that women are prompted to have children not because of the transitory low opportunity costs of childrearing but rather in response to the belief that they live in a society in which raising children and being employed are competing life course domains for women. Women who do not believe that they will be able to reenter the labor market after becoming unemployed may choose motherhood as a socially accepted biographical alternative to pursuing a career (Friedman et al. 1994; Kreyenfeld and Andersson 2014; McDonald 2000:10). Motherhood is therefore seen as a life course commitment that requires the woman to become economically dependent on a male breadwinner (or on social benefits). Thus, it is not female unemployment per se that prompts women to have children, but rather the incompatibility of work and family life, the socially defined roles of mothers, and the poor labor market prospects of women.

According to a competing hypothesis, a woman may choose to combine childrearing and paid employment, but having young children may interfere with her commitment to her job because she may feel constrained in her ability to work long hours and to travel (Del Bono et al. 2012). Moreover, potential employers in the hiring process might discriminate against women with small children (see, e.g., Correll et al. 2007). For these reasons, a career-oriented woman who is unemployed after being displaced from a job is expected to have a strong incentive to find a new job before having children and thus to postpone childbearing. Furthermore, job displacement may have long-term effects on a woman’s fertility because the loss of a job is usually accompanied by a devaluation of her firm-specific human capital. Even if the woman finds a new job, she might need time to adapt to the new environment, accumulate job-specific knowledge, and achieve the same career status and compensation level that she had in the previous job. Thus, a woman may believe that having small children would limit the amount of time she has to invest in her career. Additionally, the woman may be aware that having a child shortly after starting a new job might be perceived negatively by her employer and could therefore have negative effects on her chances of being promoted or allowed to participate in on-the-job training (Budig and England 2001).

The state of the economy is likely to be an important additional determinant in this process. In an economic downturn, when the demand for labor is relatively low, a displaced woman will tend to have more difficulties finding a new job of a quality similar to that of her previous job. Thus, if a woman prioritizes reestablishing her career, the negative effect of job displacement on her fertility could be stronger during an economic downturn than in an economic upturn. For at least two reasons, however, the negative effect of job displacement on fertility could be weaker during an economic downturn than in an economic upturn. First, a high unemployment rate and a correspondingly low probability of receiving a job offer might discourage some women from searching for a new job, and childrearing might represent an attractive biographical alternative to paid employment that is difficult to find. Second, a woman’s sociopsychological well-being might mediate the effect of a job loss on her fertility. A layoff during an economic expansion may be seen as a discretionary dismissal, which “will act as a signal of below average productivity to the displaced workers, as well as to their families and communities, and to the potential employer” (Brand 2015:362). Thus, all else being equal, a woman who has lost her job in a downturn will be less stigmatized than a woman who was displaced in an upturn. Comparing similar women who were displaced in different economic contexts—that is, in an upturn or a downturn—will shed light on the question of which of these potential mechanisms has the stronger influence on the fertility behavior of displaced women.

Our analyses are based on register data from Germany that cover an observation period of more than 20 years, from 1978 to 2003. In order to model the causal effect of job displacement, we use information on whether the woman had lost her job or changed employers because of a firm closure or a mass layoff. Firm closures and mass layoffs are beyond the control of an individual. Because they are exogenous to the decision-making of an individual, these events seem like ideal candidates for modeling the causal influences of unemployment on women’s fertility. However, several caveats must be mentioned upfront. We have no information about whether the woman was dismissed against her will or whether she left the employer in response to the mass layoff or the plant closure. Therefore, the terms “job displacement” and “job loss” used throughout this article do not necessarily reflect the conditions under which the woman exited her employment. Moreover, although we observe that, on average, women’s earnings and likelihood of being employed are lower after losing a job following a firm closure or a mass layoff, not all women experience a period of unemployment or a decline in wages after a job displacement. In addition, our analytical sample is restricted to women aged 25 or older who have been working in the same firm for at least 1.5 years. Thus, only women with a fair amount of work experience are included in our analysis. This narrow definition of the analytical sample is necessary for the causal approach we adopt in this study. As in many other studies that rely on causal approaches, we trade the causal modeling strategy against the generalizability of our results (Brand 2015). We discuss this limitation in greater detail in the concluding section of this article.

The results of our analysis suggest that the impact of a job loss is greater in an economic downturn than in an economic upturn. The women who lost a job during better economic times did not alter their birth behavior because of displacement. In contrast, the first-birth rates of the women who lost a job in an economic downturn were significantly reduced. Using a double weighting estimator, we show that these results are not driven by changes in the composition of displaced women over the business cycle.

The contribution of this investigation is threefold. First, we answer the call for more causal analysis in fertility research. Although this method is frequently used by economists, it is not yet widely diffused in demographic research. Second, we use large-scale register data combined with firm information. Many previous studies have modeled employment and fertility, but those studies have often relied on small-scale surveys and included no firm-level information. With our data, we are able to generate robust results based on highly reliable administrative data. Third and most important, we provide evidence that the effects of job displacement differ depending on the economic conditions in which the job loss occurs.

## Background

A number of macro-level studies have shown that adverse economic conditions, measured by national unemployment rates, lead to a decline in period fertility (Adserà 2011; Goldstein et al. 2013; Sobotka et al. 2011). However, micro-level analyses of the relationship between unemployment and fertility have provided rather mixed evidence (Andersson 2000; Gutiérrez-Domènech 2008; Kravdal 2002; Kreyenfeld and Andersson 2014; Matysiak and Vignoli 2013; Özcan et al. 2010; Pailhé and Solaz 2012; Schmitt 2012; Vignoli et al. 2012). Many of these micro-level studies used event-history techniques, with unemployment included as a time-varying covariate in first- and higher-order birth models. Although the results of these models indicate that male unemployment leads to a postponement of the first birth (Gutiérrez-Domènech 2008; Pailhé and Solaz 2012; Schmitt 2012), they do not appear to show that female unemployment has an impact on birth dynamics (Matysiak and Vignoli 2008; Vignoli et al. 2012). This is particularly the case for (western) Germany, where women’s unemployment has been found to have no effect (Kreyenfeld and Andersson 2014) or even a positive effect on first-birth risks (Özcan et al. 2010).

The drawback of the aforementioned studies is that they were unable to account for the possible self-selection of family-oriented women into the group of unemployed women. Unemployed women may differ in many respects from the employed population. In particular, a family-oriented woman who loses her job and becomes unemployed may be less prone to search for a new job because she anticipates that she will soon start a family and will subsequently withdraw from the labor market. Although such a bias may exist for any study that tries to examine the causal impact of female unemployment on birth behavior, it is of particular importance in a male breadwinner regime like that of Germany. In such a context, being a mother and pursuing a career are usually conceptualized as two mutually incompatible life domains. In this regime, female employment is often not perceived as a prerequisite for having children. Instead, women may withdraw from the labor market in response to adverse employment conditions, and embrace the biographical alternative of motherhood (Friedman et al. 1994; McDonald 2000).

In the economic literature, several studies have focused on mass layoffs and plant closures instead of individual unemployment spells when investigating the causal impact of job loss on individual outcomes. The advantage of this approach is that it does not suffer from potential endogeneity to the same extent as approaches that rely on individuals providing information about their employment and unemployment spells. The crucial assumption is that individuals affected by a mass layoff or a plant closure would have preferred to continue working and that the job loss is involuntary. The main aim is to compare workers who lost a job due to a mass layoff or a plant closure with similar workers who have not been affected by such an event. Many studies have applied matching techniques to ensure that the control group is similar to the group of displaced workers. In general, not all displaced workers enter unemployment. For example, some workers may search for a new job in anticipation of unemployment, whereas others may find a new job before they experience a spell of unemployment.

A number of studies have analyzed the long-term adverse effects of job displacement on labor market outcomes using this approach. For example, Jacobson et al. (1993) and von Wachter et al. (2007) have shown that job displacement leads to long-term earning losses among individuals who lost a job during the 1982 recession in the United States; Schmieder et al. (2010) found that this was also the case for displaced workers in Germany. Eliason and Storrie (2009) found long-term negative earnings effects and adverse effects of a job loss due to a plant closure on the labor market positions of workers in Sweden. Davis and von Wachter (2011) showed that the adverse effects of a job loss on earnings vary over the business cycle. Specifically, they found that the earning losses associated with a job loss are much greater during a recession than they are during an economic expansion.

Several recent studies have applied a similar approach to the analysis of demographic outcomes. For example, studies by Charles and Stephens (2004) and Eliason (2012) showed that the risk of divorce increases after job displacement. Sullivan and von Wachter (2009) found for the United States that mortality increases following job loss. Other studies have explored the impact of job displacement on health, well-being, and child outcomes (Brand 2008; Brand and Thomas 2014; Burgard et al. 2007). In the realm of fertility research, Del Bono et al. (2012) used Austrian register data to study the effects of displacement on fertility among women and men who lost a job between 1990 and 1998. They found that displacement after a plant closure significantly reduced (by 5 % to 10 %) the total fertility of women and that the effect was driven by women in white-collar jobs with high earnings and steep pre-displacement wage growth. The analysis further showed that job displacement among men also reduced fertility but that the effect did not vary depending on the men’s earnings. Thus, it appears that the effect of male displacement on fertility works through an income effect, whereas the effect of female displacement on fertility also works through an employability effect. In a related study, Del Bono et al. (2015) provided evidence that unemployment as such has no effect on fertility decisions but that job displacement leads to reduced fertility among female workers. Using Finnish register data, Huttunen and Kellokumpu (2015) analyzed the impact of job loss on fertility among women and men who were displaced between 1990 and 1993. In line with the results for Austria, they found a significant drop in fertility after job displacement among women. They reported a 1.8 % decrease in fertility 11 years after the job loss and a 4 % decrease in fertility in the year immediately following the job displacement. However, unlike the Austrian results, their findings did not show that job displacement among men led to lower fertility.

The goal of this study is first to estimate the causal effect on fertility of being displaced from a job due to a mass layoff or a plant closure and then to analyze whether a job loss during a recession is more likely than a job loss during an economic upswing to discourage a woman from having a first child. We follow a strategy similar to those used by Del Bono et al. (2012) and Huttunen and Kellokumpu (2015): we compare the birth behavior of a treatment group who experienced a mass layoff or a plant closure with the behavior of a control group who did not experience a mass layoff. Del Bono et al. (2012) and Huttunen and Kellokumpu (2015) used the mean number of births per year as their main outcome, and they included women with and without children in their analysis. However, we focus on first births only. The advantage of this approach is that our risk population is homogenous, containing only employed nulliparous women. Moreover, the labor force participation rates of women after giving birth might differ over the business cycle. This variation would introduce an additional selection problem for the comparison of displaced women in upturns and in downturns. Therefore, an investigation that includes also higher-order births is more complex than an analysis of first-birth decisions only. By looking exclusively at first-birth decisions, we can more easily establish a direct link between job displacement and subsequent birth decisions, and we can compare these decisions depending on the state of the labor market.

## Institutional Context

Because our study period covers more than two decades (1978–2003), it is important that we are aware of the policy reforms and the changes in the institutional context across time that may have affected fertility and employment behavior. During this period, female employment rates increased in western Germany. However, the institutional supports for combining full-time employment with raising children were limited. Childcare coverage for children under age 3 was less than 5 % for the entire period; and until 2005, there were relatively few places in full-time public day care for older children (Schober and Spieß 2015). However, the parental leave system changed over the period. In 1952, women became entitled to take a paid maternity leave of six weeks before and six weeks after childbirth; in 1965, this leave entitlement was extended to eight weeks. In addition, a paid parental leave scheme that granted parents six months of paid leave and income-related parental leave benefits was introduced in 1979. The benefits were equivalent to those of sick pay. In 1986, this income-related parental leave scheme was replaced by a flat-rate benefit of 600 DM (300 euros) per month, and the leave duration was increased to 10 months. Since then, the duration of the leave has been extended several times. The most significant reform was the regulation introduced in 1992 that decoupled leave payments and the duration of the leave. Parents were granted a flat-rate benefit of 300 euros per month, with a maximum leave duration of three years. These policy reforms have changed the incentive structure for women to return to work after childbirth (Gangl and Ziefle 2009; Schönberg and Ludsteck 2014) and—importantly for our study—to search for a new job after being affected by a plant closure. In addition to looking at the different responses to mass layoffs depending on the business cycle, we explore the question of whether the effects we find differ in the pre- and the post-reform periods.

## Data and Methods

We use the weakly anonymized version of the Biographical Data of Selected Social Insurance Agencies in Germany (BASiD: *Biografiedaten ausgewählter Sozialversicherungsträger in Deutschland*) provided by the Institute for Employment Research (IAB). These data contain the administrative records of individuals born between 1940 and 1992 who had made any public pension contributions up to 2007. To identify mass layoffs and plant closures, we linked our data to the Establishment History Panel (BHP) and its two extension files on firm entries and exits, and on worker flows provided by the IAB (for the discussion of the data, see Hethey and Schmieder 2010; Hochfellner et al. 2012).

One advantage of using the BASiD data is the richness of the employment histories found in the data. Another major advantage of using these data is that they cover a long observation period with several business cycles. Despite these advantages, we should mention a few caveats before we illustrate our sample construction. First, fertility records are stored reliably for women but not for men (for a validity analysis of the data, see Kreyenfeld and Mika 2008). Second, the birth histories of foreigners are not fully captured. Therefore, we restrict our investigation to women with German citizenship. Third, as in most other types of administrative data, civil servants and the self-employed are not included in BASiD. However, this limitation is not relevant in our application because civil servants and the self-employed are not affected by the treatment, as defined by displacement due to a mass layoff. Finally, the data do not contain any household-level characteristics or information on partner(ship)s, which limits our analysis of effect heterogeneity. For example, we cannot address the question of whether the employment status or the income of a partner influenced the effect of a layoff on fertility.

### Sample

We impose sample restrictions, some of which are related to the particularities of our data, and some of which are related to the particularities of the German case. We removed eastern Germans from the sample because of the differences in the fertility and the female employment behavioral patterns in the two parts of Germany (particularly before German reunification). We analyze behavior for the period 1978–2003. We do not analyze behavior before 1978 because employment records are available only from 1975 onward, and we need information on employment behavior up to three years prior to displacement. We do not analyze behavior after 2003 because the sample becomes more selective after that point.^1^

Furthermore, we restrict the sample to women who were employed for at least 1.5 years at the same firm. This restriction ensures that we can include firm-level control variables from the year *j* – 1 because these variables are measured every year in June (see the section Treatment and Control Groups). We also omit women who worked in the agricultural or construction sector because of the high percentage of seasonal work in this sector. Women who were working at firms that never had more than five employees were also excluded because of the difficulty of defining a mass layoff for this group. We furthermore exclude women who had worked in firms with more than 2,000 employees, given that the matching procedure requires that we find comparable women in control and treatment groups. For large firms, it is simply difficult to find suitable matches. Finally, we also restrict our sample to women who were between the ages of 25 and 40 because a relatively large share of women under age 25 have not yet completed their education, and a relatively small share of women over age 40 give birth to a first child.

### Treatment and Control Groups

To build the treatment indicator, we use firm and individual information (see also Table 6 in the appendix, which describes the setup of the data based on one case). We define a woman as being treated in quarter *q*, the reference quarter, if she is employed in a firm in quarter *q* but not in quarter *q* + 1, and the firm had either a mass layoff or a plant closure. Firm data are available on a yearly basis, measured on June 30. We identify a mass layoff of a firm in year *j* if the number of employees of that firm decreased by more than 30 % either between *j* – 1 and *j* or between year *j* and *j* + 1.^2^ The control group consists of women who did not leave a firm or who left a firm that did not experience a mass layoff or closing. In this design, women who are treated in quarter *q* can be part of the control group in any quarter before *q* (conditional on fulfilling our sample restrictions with respect to age and tenure). A woman treated in *q* cannot be part of the control group in the quarters *q* through *q* + 5; she can be included only from quarter *q* + 6 onward. In the Empirical Findings section, we describe a sensitivity analysis in which we exclude women from the control group the year before they were displaced due to a mass layoff.

Table 1 reports the total number of children as well as the share of women who were childless by birth cohort and by whether the woman was ever “treated” or was part of the control group. For example, we find that among the western German 1950–1959 cohorts, the average number of children is 1.7—a figure that is very close to the cohort fertility estimate for these cohorts published by the German Statistical Office (Statistisches Bundesamt 2007). Our findings further indicate that the share of women in these cohorts who were childless is approximately 17 %, which is also in the expected range (Kreyenfeld and Konietzka 2017). Among the subsequent cohorts, the total number of children born declined and the share remaining childless increased. These trends are in line with known fertility developments in western Germany. However, for the very young cohorts, early censoring explains why a very large share of women were childless and why they had a small number of children.

**Table 1:** Fertility and labor market participation by birth cohorts of women, BASiD data and analytical sample

	Birth Cohort			
	1940–1949	1950–1959	1960–1969	1970–1976
All				
Childless (%)	12.78	16.67	21.44	39.31
Number of children	1.87	1.68	1.52	1.05
Never worked (%)	75.36	11.94	12.48	16.70
Number of observations	26,693	25,990	29,230	19,997
Analytical sample				
Treated				
Childless (%)	27.70	37.04	44.33	61.16
Number of children	1.33	1.09	0.92	0.54
Never worked (%)	65.62	38.08	38.52	37.85
Number of observations	509	1,053	1,119	605
Untreated				
Childless (%)	22.49	24.32	29.50	49.19
Number of children	1.47	1.41	1.28	0.78
Never worked (%)	66.76	37.35	39.35	39.34
Number of observations	2,494	4,075	4,956	3,025
Number of observations	3,003	5,128	6,075	3,630

*Notes:* All information is drawn from raw data without restrictions. Analytical sample: Sample used for the analysis. “Treated” includes all women who have ever been treated in the observation sample. “Untreated” includes the women who have never been treated in the observation period. “Never worked” are persons who have no employment spells in the registers between 1975 and 2007.

A comparison of the treated and the untreated cases reveals very large differences. The total number of children was much smaller for our sample of women, who, given our study design, must have been employed for some part of their life. Hence, our investigation is limited to women who had some labor market attachment before they had their first child and who did not have their first child before the age of 25. Although this group is growing in western Germany, it is not representative of the entire female population in Germany, especially of the older cohorts. In our empirical analysis, we allow for time trends in fertility behavior to ensure that our results are not driven by these general trends in employment and fertility behavior in western Germany.

### Methods

In conducting our empirical analysis, we face two major challenges. First, the women who are treated (i.e., displaced due to a mass layoff or a plant closure) may differ from the untreated women in terms of their skill level, degree of career orientation, or other characteristics. For example, compared with women who are less career-oriented, women who are more career-oriented may be less likely to be laid off and be less inclined to have a family. We refer to these differences as *first-order differences*. The effect of being displaced on fertility might therefore be biased in a research design that does not take the potential endogeneity of the displacement into account. In our research design, we use mass layoffs and plant closures as treatment-inducing exogenous events. They provide us with a quasi-natural experiment setting: after detailed lagged employment outcomes and firm characteristics are controlled for, it seems plausible to assume that an exogenous shock that hits a firm or a (local) economy and leads to a mass layoff or a plant closure is independent of individual characteristics related to a woman’s fertility decision. It is important to note that we do not regard individuals who leave a firm in the absence of a mass layoff or a plant closure as treated but rather as part of the control group.^3^ In sum, in our research design, the crucial assumption is that the treatment is conditionally independent of the fertility decision after we control for detailed individual- and firm-specific information.

Our second major challenge is that different treatment effects in periods with high and low unemployment rates can be driven by different underlying effects of experiencing a mass layoff or a plant closure. Alternatively, they could be driven by changes in the composition of the laid-off women. We refer to these latter differences as *second-order differences*. If the characteristics of the women who were laid off in an economic downturn differ from the characteristics of the women who were laid off during an economic upturn, and if the effects of job displacement on fertility outcomes vary by observed characteristics, a change in the composition of the treated women might partly explain the different treatment effects. We use a double weighting estimator to adjust for potential second-order differences and to ensure the comparability not only of the treated and the nontreated women but also of the women who are treated in an upturn and the women who are treated in a downturn. However, as we show in the Empirical Findings section, these estimates do not change much when compared with the results of a model in which we ignore these second-order differences.

We estimate the effect of having been laid off in quarter *q* on the probability of becoming pregnant within a given time interval *t* after quarter *q*, *y*_*iqt*_ = P(*preg*_*iqt*_ = 1). Our baseline specification is a linear probability model (LPM):

1 $$ {y}_{iq t}={\upbeta}_0+\updelta {D}_{iq}+{\mathbf{X}}_{iq}{\boldsymbol{\upbeta}}_1+{\mathbf{X}}_{fq}{\boldsymbol{\upbeta}}_2+{\upvarepsilon}_{iq t}. $$

Time interval *t* refers to *t* years after quarter *q*. The treatment indicator *D*_*iq*_ is a dummy variable of 1 if individual *i* has been laid off in quarter *q* due to a mass layoff or a plant closure. δ is the coefficient of interest, and it corresponds to the causal impact of being laid off on the probability of becoming pregnant*.* **X**_*i*_ and **X**_*f*_ are vectors of control variables on, respectively, the individual level and the firm level. **β**_1_ and **β**_2_ are the corresponding coefficient vectors, and β_0_ is the intercept. We estimate Eq. (1) simultaneously for all quarters in our observation period. As we described earlier, one woman can enter with several observations. Standard errors are clustered at the individual level.^4^

To account for differences in δ between economic downturns and economic upturns, we introduce two treatment effects: one for mass layoffs and plant closures during periods with relatively high unemployment rates, and one for mass layoffs and plant closures during periods with relatively low unemployment rates:

2 $$ {y}_{iq t}={\upbeta}_0+{\updelta}_1{D}_{iq}\left({U}_q\right)+{\updelta}_2{D}_{iq}\left(1-{U}_q\right)+\upkappa {U}_q+{\mathbf{X}}_{iq}{\boldsymbol{\upbeta}}_1+{\mathbf{X}}_{fq}{\boldsymbol{\upbeta}}_2+{\upvarepsilon}_{iq t}. $$

The binary indicator *U*_*q*_ is 1 in periods with high unemployment rates and 0 otherwise. This implies that δ_1_ captures the impact of being laid off in an economic downturn and that δ_2_ captures the impact of being laid off in a period with relatively good labor market prospects.

In addition to the LPMs, we apply more flexible IPW estimators and present heterogeneous effects based on split samples (high and low unemployment rates). For constructing the weights, we estimate logit models for the probability of being displaced due to a mass layoff or a plant closure. Using these models, we estimate the individual probability of being treated—that is, the propensity scores, ${\widehat{p}}_{iq}$. Our main interest is in the estimation of the average effect of being treated for the sample of treated women (ATT). Therefore, the weights for the treated women are 1 (*w*_*iq*_ = 1), and the weights for the nontreated women are ${w}_{iq}={\widehat{p}}_{iq}/1-{\widehat{p}}_{iq}$. For the estimation of the ATT, the outcome variable, *y*_*iq*_, is regressed on the treatment dummy variable, *D*_*iq*_, applying the individual weights, *w*_*iq*_ (see, e.g., Morgan and Winship 2015). We report standard errors obtained by bootstrapping, which we perform by resampling at the person level.

To address the second-order differences, we apply a double IPW estimator. The idea behind this procedure is to tailor a sample of treated women in good labor market conditions that resembles the sample of treated women in a downturn, and then to use these samples (of women in a downturn and women in an upturn) to estimate separate treatment effects while adjusting for any compositional differences of the treated population. We use this procedure to calculate the weights twice. The weights of the first step are based on a logit model estimated with all *treated* women. The dependent variable is *treated in a downturn*. These weights are used to keep constant the composition of treated women in a downturn. The weights of the second step are based on logit models for being displaced due to a mass layoff, separately for the samples in times of high and of low unemployment rates, *and* using the weights of the first step. Any differences between the treatment effects of these two samples will be driven by differences in the underlying effects of experiencing a layoff (as opposed to differences in characteristics). For similar approaches to comparing the effectiveness of various labor market programs over time, see Lechner and Wunsch (2009) and Rinne et al. (2013), who used matching methods; and see Heinrich and Mueser (2014), who applied IPW estimators.

### Variables and Descriptive Statistics

At the individual level, we control for age with a categorical variable (ages 25–27, 28–30, 31–33, 34–36, 37–38; and ages 39–40 as reference category). We also control for tenure (with a dummy variable showing whether the individual was employed at the firm for more than 2.5 years), occupation (with a set of dummy variables), sector of the last job (with a set of dummy variables), and earnings in the quarters of the past three years. To measure the quarterly earnings, we calculate the percentile position of each woman within the earnings distribution in the given year. Thus, our earnings measure indicates the position in the earnings distribution, ranging from 0 to 100. Using the relative position within the yearly earnings distribution instead of the real earnings in euros has the advantage of providing a greater degree of comparability across years. At the firm level, we control for the number of employees (11–50, 51–250, more than 250; with 4–10 as reference); the earnings distribution (using the within-firm 25th, 50th, and 75th percentiles to define the position (percentile) of these wages within the overall earnings distribution of the corresponding year); and the shares of employees who were under age 30, aged 30–49, and aged 50 or older. We also include the shares of the workers in each firm who are women and who are low-skilled. These firm-level characteristics are measured on June 30 of each year. A mass layoff usually changes firm-level characteristics, such as wage structure, and the inclusion of these endogenous characteristics would bias the coefficient of interest. Using lagged variables solves this problem. Thus, we include the firm-level characteristics measured in year *t* – 1. In contrast, the individual characteristics are measured in the corresponding quarter *q*. We also insert the calendar year as a linear and quadratic variable into the equation to control for changes in the general trends in fertility behavior over time. The time is measured in years, with the year 1978 corresponding to *t* = 1. To control for seasonal patterns in job destruction and employment behavior, we include quarterly dummy variables that capture seasonal effects.

**Fig. 1:**
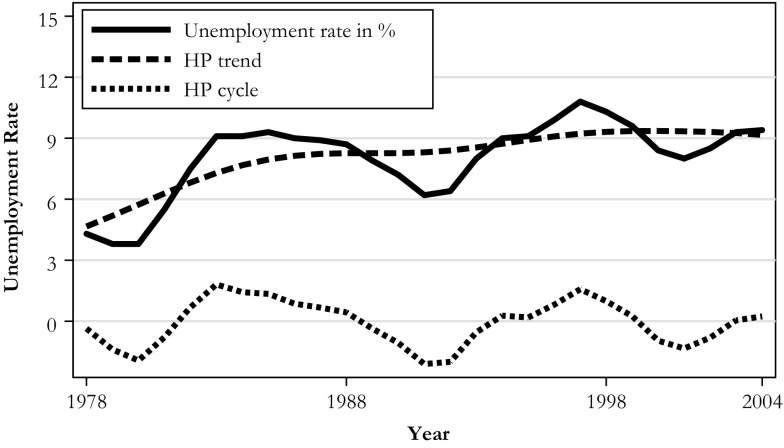
Business cycle in western Germany (1978–2004). Hodrick-Prescott filtered trend (HP trend) and deviation of the unemployment rate from the trend (HP cycle). *Source:* Data from the Federal Employment Agency

To investigate whether the effect of displacement on fertility varies with the business cycle, we compare women who were treated when the annual unemployment rate was low with women who were treated when the unemployment rate was high.^5^ However, given our long observation period, the years in which the unemployment rate was relatively low may have other characteristics in common. Thus, a business cycle indicator (solely) based on the unemployment rate may reflect period effects rather than the economic conditions (cyclical variation). This possibility is especially relevant in our case: there might have been time trends in the fertility and labor supply behavior of the women in our sample because the unemployment rate in Germany has been increasing over time. To split the observed unemployment rate into its trend and its cyclical component, we apply the Hodrick-Prescott filter in the same manner as van den Berg et al. (2006). Figure 1 depicts the observed unemployment rate, its trend, and its cyclical component (i.e., the difference between the unemployment rate and its trend). Our indicator of an economic downturn is a dummy variable of 1 if the cyclical component is positive—that is, if the unemployment rate is higher than the general trend.

In Table 2, we present descriptive statistics for selected characteristics of women who were treated and not treated for periods with high and low unemployment rates. Table 2 suggests that the treated women differ from the control group in terms of both their individual characteristics (such as previous tenure and earnings) and the characteristics of their employer (such as firm size). After we use IPW to account for the differences between the treated and the control groups, we find that the differences are very small and are not statistically significant. Table 2 also suggests that the women who were treated in a downturn differ only slightly from those who were treated in an upturn. For example, compared with the women who were treated in a boom, the women who were treated in a downturn were employed by lower-paying firms. By applying double inverse probability weighting, we account for these (second-order) differences.

**Table 2:** Descriptive statistics

	Downturn			No Downturn		
		Untreated^a^			Untreated^a^	
	Treated	Unweighted	Weighted	Treated	Unweighted	Weighted
Individual Characteristics						
Tenure > 2.5 years	0.672	0.790***	0.671	0.714	0.814***	0.716
Wage quarter – 1	49.493	53.314***	49.439	47.867	52.663***	47.810
Wage quarter – 7	0.971	0.982*	0.971	0.978	0.983	0.980
Wage quarter – 12	0.876	0.912***	0.876	0.904	0.922	0.904
Firm Variables						
Wage 25th percentile	39.504	43.035***	39.445	41.195	43.332**	41.135
Wage median	51.517	56.191***	51.462	53.638	56.846***	53.600
Wage 75th percentile	63.989	70.18***	63.941	67.379	71.186***	67.377
Share of employees < age 30	0.350	0.342	0.350	0.354	0.340*	0.354
Share of employees ≥ 30 and < 50	0.487	0.479	0.486	0.484	0.484	0.484
Share of employees ≥ age 50	0.163	0.179***	0.163	0.162	0.176***	0.163
Share of female workers	0.582	0.575	0.583	0.580	0.574	0.581
Share low qualified	0.211	0.226^†^	0.211	0.215	0.230^†^	0.215
Firm size 4–10 employees	0.237	0.137***	0.238	0.243	0.132***	0.242
Firm size 11–50 employees	0.259	0.194***	0.259	0.255	0.199***	0.256
Firm size 51–250 employees	0.249	0.247	0.249	0.238	0.254	0.237
Firm size 251–1,999 employees	0.255	0.423***	0.253	0.264	0.415***	0.265
Number of Observations	643	60,066		614	53,735	

*Notes:* The table displays the means of selected control variables, separately for treated and untreated and by downturn and upturn. The individual characteristics are measured in the corresponding reference quarter, and the firm-level characteristics are measured in the calendar year before the reference quarter. For the control group (untreated), the unweighted and the weighted means are presented. The weights (single weighting) are explained in the text. Table S1 in Online Resource 1 contains the means of all control variables used in the analysis. The estimations from the logit model for constructing the weights are reported in Table S5 in Online Resource 1.

^a^Significance of differences (*t* test): ^†^ *p* ≤ .10; *p ≤ .05; **p ≤ .01; ***p ≤ .001

## Empirical Findings

### Employment Dynamics After Firm Closure

**Fig. 2:**
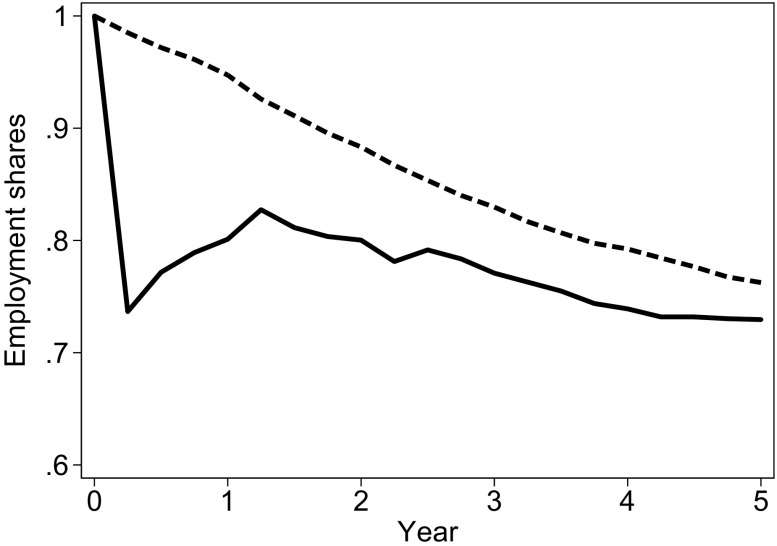
Employment shares by time since the displacement for the treated group (solid line) and time since the “reference quarter” for the control group (dashed line). Weighted shares in full-time employment. The inverse probability weights (IPW) are used as described in the text. Year 0: Year of displacement. Number of observations: 115,273 quarterly spells of 8,179 individuals. 1,262 treated spells. *Source:* Own calculations based on BASiD data

We are interested in examining the causal impact of an involuntary job loss on the probability of having a first child. In this context, we interpret a job loss due to a mass layoff or a plant closure—the “treatment”—as an exogenous shock to the employment career of the treated woman. We start with the investigation of these effects on employment. Figure 2 displays the shares of the treated women and of the weighted sample of the untreated women who were employed.^6^ Because of our sample selection criteria, all the women were employed during the six quarters before the reference quarter *q*. This weighting approach ensures that the shares of the treated and the nontreated women who were employed are close to identical between 1.5 and 3 years before the reference quarter (not displayed).

Even in the absence of a mass layoff or a plant closure in quarter *q*, the share of women who are employed decreases over time. Female employment may decline for a number of reasons. For example, a woman may lose her job in the absence of a mass layoff or a plant closure, or she may be affected by a mass layoff or a plant closure in later periods. Alternatively, she may leave employment voluntarily. Our findings show that a woman who was displaced from a job had a substantially lower probability of being employed in the years after the displacement. In line with evidence from other countries, we find that the chances of being employed were lowest in the period directly after the layoff (Del Bono et al. 2012; Huttunen and Kellokumpu 2015). The difference between the treated and the nontreated women decreased from around 25 percentage points in the first quarter after displacement to approximately 3 percentage points five years after the job loss. All the estimated differences are statistically significant at the 0.1 % or the 1 % level, showing that involuntary job loss has short- and medium-run negative effects on the employment outcomes of the treated women.

### Birth Dynamics After Firm Closure

**Fig. 3:**
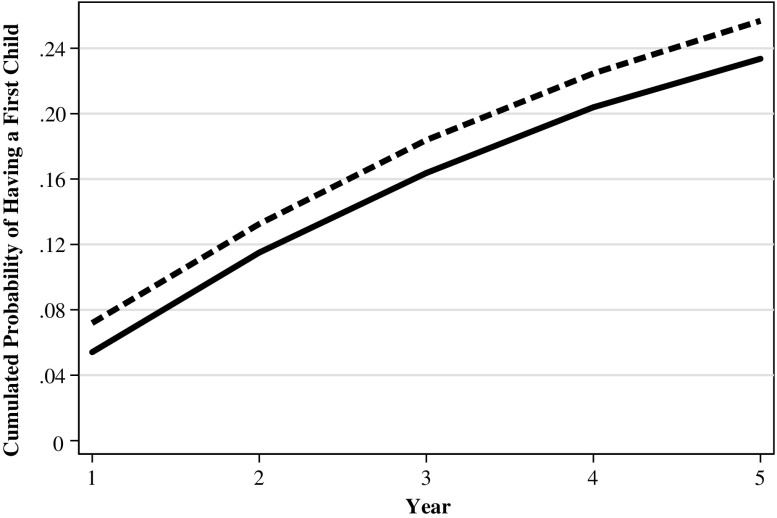
Cumulated first pregnancy probability by time since the displacement for the treated group (solid line) and time since the “reference quarter” for the control group (dashed line). *Source:* Own calculations based on BASiD data

Figure 3 plots the unconditional probability of having become pregnant up to year *t* after the displacement for the treated and the control group. The figure provides descriptive evidence that a woman who lost a job due to a mass layoff or a plant closure has a lower probability of having given birth to a first child and that this difference was relatively constant over the five years after the job displacement.

**Fig. 4:**
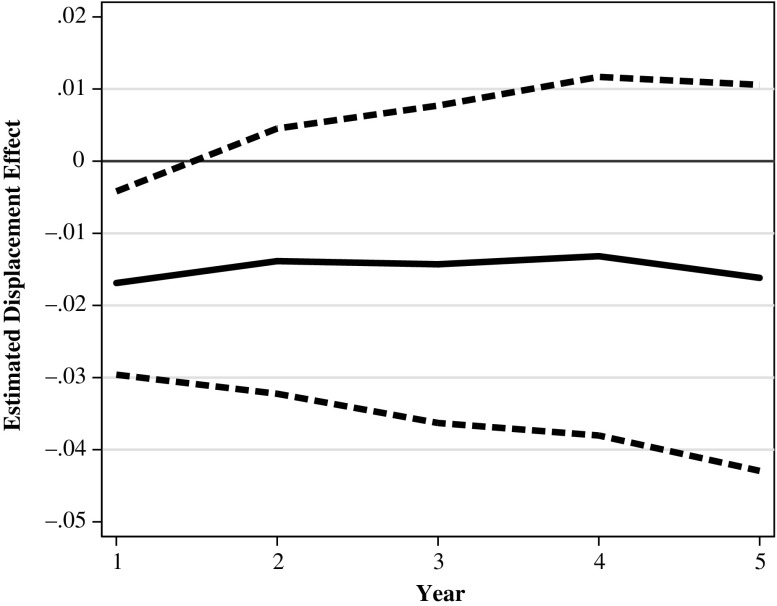
The estimated displacement effect on the cumulated pregnancy probability by duration since the displacement/time since the reference quarter (solid line) and the 95 % confidence bounds (dashed line). Average marginal effects from a linear probability model. *Source:* Own calculations based on BASiD data

Can differences in observed characteristics explain these raw differences displayed in Fig. 3? After controlling for observed characteristics, we find that this is not the case (Fig. 4 and Table 3, Models A and B). Rather, the results are in line with those shown in Fig. 3. More specifically, we get a constantly negative point estimate for the treatment on the cumulated probability, which suggests that the probability of becoming pregnant decreased between 1.2 and 1.7 percentage points in the years after the displacement using the LPM and the IPW estimators. Compared with untreated women, the cumulated pregnancy probability for treated women is reduced by 23.6 % in the first year, by 7.6 % in the third year and by 6.2 % in the fifth year.^7^ However, the coefficient is significantly different from 0 only for the year immediately after displacement. The results of the LPM and the IPW estimators are very similar.

**Table 3:** Effect of layoffs on fertility: Average effects

Year After Layoff	Model A: LPM Treated	Model B: IPW Treated	Number of Observations
1	−0.017**	−0.017**	115,058
	(0.006)	(0.007)	
2	−0.014	−0.014	109,027
	(0.009)	(0.010)	
3	−0.014	−0.014	102,651
	(0.011)	(0.012)	
4	−0.013	−0.012	96,494
	(0.013)	(0.013)	
5	−0.016	−0.015	90,560
	(0.014)	(0.014)	

*Notes:* Dependent variable is cumulated first-birth probability. Model A: OLS regression of the linear probability model. The full model for year 1 after the layoff is reported in Table S6 in Online Resource 1. Model B: Inverse probability weighting (IPW) estimation. For the IPW estimators, the standard errors are bootstrapped with 500 replications. Controlled for the variables listed in Table S1 in Online Resource 1.

**p ≤ .01

### The Fertility Response to Displacement Over the Business Cycle

**Fig. 5:**
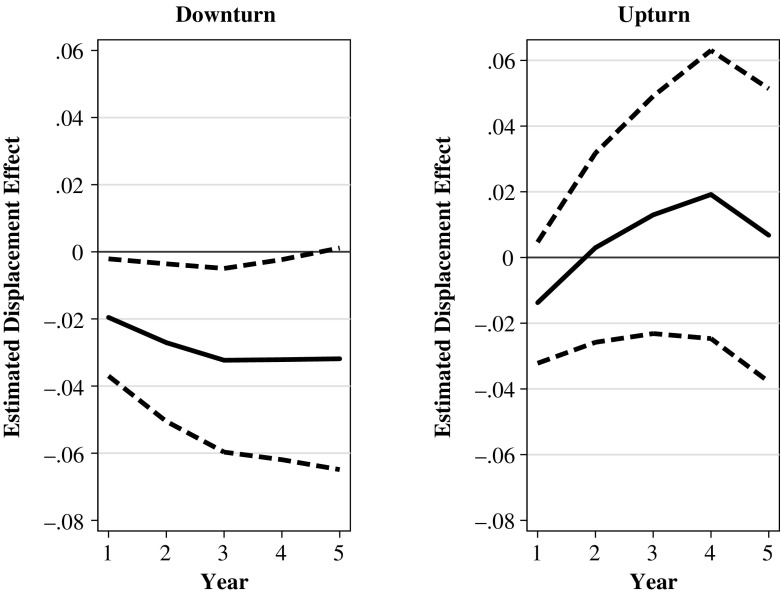
The estimated displacement effect on the cumulated pregnancy probability by duration since the displacement/time since the reference quarter and by upturn and downturn (solid line). 95 % confidence bounds (dashed line). Average marginal effects from a linear probability model. Downturn: The unemployment rate is greater than the unemployment trend. Upturn: The unemployment rate is smaller than or equal to the unemployment trend. Source: Own calculations based on BASiD data

The primary goal of our study is to investigate whether the effects of displacement on fertility outcomes differ between women who were laid off in a downturn and women who were laid off in a boom. Figure 5 depicts graphically our main findings by plotting the ordinary least squares (OLS) coefficients from Model C (Table 4). We find a significant negative impact of having been laid off in a recession on the cumulated probability of becoming pregnant during the first five years after the treatment. Our results suggest a 2 percentage point decrease in the probability of becoming pregnant in the year immediately after the displacement. The displacement effect increases in absolute size to a 3.3 percentage point decrease in the probability of becoming pregnant in the three to five years after the displacement. All the coefficients of Model C are statistically significant at the 5 % level. Although our findings indicate that layoffs during times of high unemployment affect fertility, they do not show that layoffs during times of low unemployment have significant effects on fertility.

**Table 4:** Effect of layoffs on fertility by business cycle

	Model C: LPM		Model D: IPW		Model E: Double IPW	
Year After Layoff	Treated in Downturn	Treated in Upturn	Treated in Downturn	Treated in Upturn	Treated in Downturn	Treated in Upturn
1	−0.020*	−0.014	−0.020*	−0.013	−0.019*	−0.019*
	(0.009)	(0.009)	(0.009)	(0.010)	(0.009)	(0.010)
2	−0.027*	0.003	−0.027*	0.003	−0.027*	−0.005
	(0.012)	(0.015)	(0.012)	(0.015)	(0.012)	(0.016)
3	−0.033*	0.014	−0.032*	0.013	−0.032*	0.008
	(0.014)	(0.019)	(0.014)	(0.018)	(0.014)	(0.022)
4	−0.033*	0.024	−0.032*	0.021	−0.032*	0.032
	(0.015)	(0.022)	(0.016)	(0.023)	(0.016)	(0.031)
5	−0.033*	0.012	−0.032^†^	0.010	−0.035*	0.009
	(0.017)	(0.023)	(0.018)	(0.023)	(0.018)	(0.031)

*Notes:* Dependent variable is cumulated first-birth probability. Model C: OLS regression of linear probability model for the pooled sample including interaction terms. The full model for year 1 after the layoff is reported in Table S6 in Online Resource 1; Model D: Inverse probability weighting (IPW) estimation for separate samples (upturn/downturn); Model E: Double IPW estimation for separate samples (upturn/downturn). In the double IPW, we cannot control for time trends because, by definition, the upturn and the downturn take place at different times. Therefore, the point estimates differ slightly from those reported in Model D. For the IPW estimators, the standard errors are bootstrapped (500 replications). See Table 3 for number of observations.

^†^ *p* ≤ .10; *p ≤ .05

The separate IPW estimations do not differ qualitatively from these results. We find no significant impact during periods of low unemployment (Table 4, Model D). In contrast, after treatment in times of high unemployment, the effect is negative and is statistically significant at the 5 % level for the first four years and at the 10 % level in the fifth year. The point estimate increases from a 2 percentage point decrease in the probability of having a first child in the first year after the displacement to a 3.2 percentage point decrease in the fifth year after the job loss. Notably, the sizes of these results are about the same as the effect sizes that are estimated based on the LPM. We find that economic upturns and economic downturns had significantly different effects on the first-birth rates after three and four years.^8^ This suggests that compared with the women who lost a job due to a mass layoff during an economic boom, the women who lost a job due to a mass layoff during a recession were less likely to have given birth to a first child in the medium term. Among the women in the control group who were employed in a downturn, we observe that 7.2 % became pregnant within one year after the reference quarter. The IPW effects indicate a decrease of 2.0 percentage points, which corresponds to a relative decrease of 27.7 % after one year. After five years, 25.7 % of the women from the control group who remained employed in a downturn had experienced a first birth. The IPW effect of −0.032 corresponds to a relative decrease in first-birth probabilities of 12.8 %.

### Accounting for Changes in the Composition of the Treated Women Over the Business Cycle

To investigate whether our results are driven by changes in the composition of women laid off in an economic downturn compared with the composition of women laid off during an economic upturn, we use a double weighting approach. Our reference population consists of treated women who were displaced due to a mass layoff during a downturn.

Accounting for differences in the composition does not change our main results. Although we observe a significant reduction in the cumulated probability of having had a first birth in the first year after treatment, the estimated effects from two years onward are negative and significant for layoffs in downturns, and are statistically insignificant and close to 0 or positive for layoffs in times of low unemployment (Table 4, Model E). This clearly indicates that differences in the effects between treatment in an economic downturn and treatment in an economic upturn are not driven by changes in the composition of the treated women.

### Potential Violations of Our Identification Strategy

In our empirical approach, we investigate the impact of a job loss on the fertility behavior of the displaced women. In addition, some women might have anticipated a mass layoff and may therefore have postponed or accelerated the transition to the first child. This could have an important impact on the validity and the interpretation of our estimates.

To evaluate this potential violation of our identification strategy, we redefine our treatment. We change the sample from the main analysis in two respects: first, we include women who were mothers in quarter *q* (but not in the year before); and second, we redefine the treatment as having worked at a firm that had a mass layoff or closed down within the year after quarter *q* (Table 5, Model F). We find that in a recession, when a mass layoff might be anticipated, first-birth probabilities were slightly lower, by 0.6 percentage points. This rather small effect is statistically significant at the 10 % level. We find no evidence of an anticipation effect in times of low unemployment. This evidence suggests that our estimates are not biased upward by anticipation effects of mass layoffs.

**Table 5:** Effect of layoffs on fertility by business cycle, controlling for major reforms of the parental leave system

	Model F Anticipation		Model G: LPM With Reform Effects (1986) Treated		Model H: LPM With Reform Effects (1992) Treated	
Year After Layoff	In Downturn	In Upturn	In Downturn	In Upturn	In Downturn	In Upturn
−1	−0.006^†^	0.000	––	––	––	––
	(0.003)	(0.003)				
1	––	––	−0.003	0.005	−0.006	0.000
	––	––	(0.015)	(0.018)	(0.012)	(0.015)
2	––	––	−0.025	0.006	−0.028^†^	0.002
	––	––	(0.019)	(0.022)	(0.016)	(0.019)
3	––	––	−0.047*	0.000	−0.036^†^	0.011
	––	––	(0.021)	(0.025)	(0.019)	(0.022)
4	––	––	−0.041^†^	0.017	−0.028	0.024
	––	––	(0.023)	(0.028)	(0.021)	(0.024)
5	––	––	−0.043^†^	0.003	−0.033	0.011
	––	––	(0.024)	(0.029)	(0.022)	(0.025)

*Notes:* Dependent variable is cumulated first-birth probability. Model F: Treatment: Working at a firm with a mass layoff within the following year, and the outcome is having a child in the year before the mass layoff; the sample consists of the main analysis sample and those women who became pregnant in the year before the mass layoff. Data contain 15,237 spells of women at a firm with a mass layoff in the following year, and 108,561 spells of women at a firm without a mass layoff in the following year. Model G: Accounting for changes in the parental leave system in 1986. In this model, we allow for a shift in the treatment effect after the reform, independent of the status of the business cycle. Model H: Similar to Model G, but accounting for changes in the parental leave system in 1992. See Table 3 for number of observations.

^†^ *p* ≤ .10; *p ≤ .05

Another potential source of violation of our identification strategy is changes in the parental leave system over time. We address this potential problem by reestimating our models by allowing for a different effect of being displaced due to a mass layoff or a plant closure before and after the two most relevant parental leave reforms, which were enacted in 1986 and 1992. The results are reported in Table 5, Models G and H. We find that although the impact is lower in the first year after displacement, the other coefficients are qualitatively stable, with negative point estimates between −0.025 and −0.047 for the reform in 1986 and between −0.028 and −0.036 for the reform in 1992/1993 for periods of high unemployment, and positive point estimates for periods of low unemployment. We lose statistical power in this step of the analysis, and one-half of the coefficients lack statistical significance at the 10 % level. Nevertheless, we are not worried about the validity of our results because our coefficients for the second to the fifth year after the job displacement are all negative and are statistically significant at the 10 % level (year 2) and at least at the 5 % level (years three to five) after we use the continuous cyclical component of the unemployment rate instead of the binary indicator for low and high unemployment rates; see Online Resource 1, Table S2, Models A2 and A3.

We conducted several additional robustness checks that include models based on continuous unemployment rates instead of binary indicators and models based on the gross domestic product to define the status of the business cycle. We present the corresponding results in Online Resource 1. Overall, our results are robust with respect to alternative specifications.

## Conclusions

In this study, we analyzed the effects of job displacement on women’s first-birth rates, and the variation in these effects over the business cycle using rich administrative data for Germany spanning more than 20 years. We used mass layoffs and plant closures to estimate the impact of involuntary unemployment on fertility in the short and the medium term (up to five years after displacement). The main finding from our analysis is that job displacement has adverse effects on the likelihood of having a first birth, and the impact is stronger in an economic downturn than in an upturn. Compared with the women who lost a job in times of low unemployment, the women who lost a job in times of high unemployment experienced a significant reduction in the probability of having given birth to a first child even five years after the job loss. These results are not driven by changes in the composition of the displaced women over the business cycle.

The results show that in a downturn, first-birth rates decreased by approximately 28 % in the first year after displacement, and by 13 % in the fifth year after the job loss. On average, fertility decreased by approximately 24 % in the first year and of 6 % in the fifth year, with the effect being statistically significant for the first year only. Comparing our estimates with previous findings from Del Bono et al. (2012) and Huttunen and Kellokumpu (2015), however, is not straightforward. Although these authors applied a similar econometric approach, they investigated the impact of job displacement on the number of births and thus did not estimate the different birth orders separately. However, Del Bono et al. (2012) provided results for the subgroup of childless women over age 24, which is comparable with our sample. For this group, they found a decrease of 0.027 in the number of births three years after displacement. This figure corresponds to an 11 % reduction in first-birth rates. Our results are similar in size: we find an (insignificant) reduction of approximately 8 % three years after displacement.^9^

Our findings suggest that the economic context plays an important role in the impact of a job displacement on fertility behavior. We argue that job displacement is more detrimental for fertility choices in a downturn because women tend to have more difficulties finding a new job that is similar in quality to their previous job in a recession than in an economic boom. An alternative explanation could be that an economic downturn creates a context in which displacement is less selective. The assumption is that when workers experience job displacement in an economic downturn, the chances are lower than they are in an economic upturn that these workers have been dismissed for reasons that might also be relevant for their outcomes—in our case, their fertility decisions. If selection into job displacement is very different in downturns than in upturns in our setting, we would expect to generate different estimates for the treatment effects when applying the double weighting estimator to correct for differences in very detailed and informative observed characteristics. As we described earlier, it turns out that the point estimates are very similar to estimates produced by an analysis that does not correct for these differences in the observed characteristics. Given this evidence, we do not believe that a different selection into job displacement in economic upturns and downturns drives our main results.

Our analysis responds to the growing call for more causal analysis in fertility research. With our study, we provide a “clean estimate” of the effect of job displacement on fertility. Although many prior studies suffered from the inability to control for the selection into unemployment, we overcome this problem by using a mass layoff as an exogenous shock to the employment career of a woman. Despite the attractiveness of this approach, we need to acknowledge that upon closer inspection, our estimate is not as “clean” as we would like it to be.

First, we did not measure unemployment. A plant closure or a mass layoff may lead to a woman becoming unemployed, but it may also simply result in a woman changing employers without ever experiencing a spell of unemployment. Thus, we did not produce clear-cut estimates of the effect of unemployment on fertility. Instead, our estimates reflect both the effects of job loss and the challenges of settling into a new job.

A second and related issue is that a mass layoff or a plant closure may be less detrimental for a woman’s life course than becoming unemployed after an involuntary dismissal. Although a job displacement may be expected to have a negative effect on a woman’s income and well-being, a dismissal in conjunction with a mass layoff may be regarded as a collective fate and may thus be less damaging to the woman’s psychological well-being than being fired. Although our outcome is fertility and not health or well-being (see, e.g., Burgard et al. 2007), there may be indirect loops that link well-being and fertility, especially through the effect of well-being and health on partnership stability.

Third, because our analysis focuses on a subset of the German population, our results may not be generalizable to the entire population. We had to limit our sample to work-committed women older than 24. From a social policy perspective, we neglected the more vulnerable groups who experience unemployment at younger ages and who are subject to discontinuous employment careers. Moreover, the restrictions on the sample reduced its size, which in turn limited our ability to conduct in-depth investigations. Although we were able to measure the average impact of job displacement over the business cycle, the size of our analytical sample did not allow us to investigate the potential heterogeneity of these effects with respect to, for example, the woman’s skill level, occupation, or employment sector. Examining this kind of effect heterogeneity could help to shed more light on the underlying mechanisms of the average effects found in our study.

Our results support similar causal investigations on the same topic, but they seem to challenge prior empirical findings that modeled the relationship between unemployment and fertility in an event-history framework. Although many prior studies showed no effect or even a positive effect of women’s unemployment on first-birth rates (Andersson 2000; Gutiérrez-Domènech 2008; Kreyenfeld and Andersson 2014; Kreyenfeld et al. 2012; Matysiak and Vignoli 2008; Özcan et al. 2010; Pailhé and Solaz 2012; Schmitt 2012; Schröder 2010; Vignoli et al. 2012), we found that a careful causal analysis suggests that adverse employment conditions lead to first-birth postponement among women, even in the conservative welfare state of western Germany. Does this mean that the association between female unemployment and the birth risks found in the abovementioned studies simply reflect the selectivity of the unemployed population?

In order to answer that question, we have to return to the selection of our analytical sample. The approach we adopted covers a subsection of the western German population. Our main restrictions in selecting the sample were that the women had to be aged 25–40 and had to have worked for the same firm for at least 1.5 years. Thus, we focused on a selected and work-committed subpopulation. Women who are under age 25, have a low level of attachment to the labor market, or have a less continuous employment career may respond differently to unemployment and other types of labor market uncertainties. Prior studies that investigated the interaction effects between unemployment, age, and fertility lend support to this claim. Based on Danish register and German survey data, Kreyenfeld and Andersson (2014) found that unemployment at young ages increases first-birth rates but has the reverse effect at higher ages. Rendall et al. (2009) also reported interaction effects of unemployment, age, and fertility for the UK and France.

From this perspective, the results from our study appear to be more in line with those of standard event-history studies showing that work-committed women delay childbearing if they are exposed to labor market uncertainties. In western Germany, this group of women has been rather small, at least until recently. Therefore, a careful causal analysis or an investigation by population subgroup was needed to carve out this effect. Germany has enacted major policy reforms since 2005. The expansion of public day care for children under age 3 and the parental leave benefit reform of 2007 greatly improved women’s options for combining work and family. In light of the rising levels of female labor force participation in western Germany and in many other industrialized countries, the group of work-committed women is increasingly representative of the female population.

### Electronic supplementary material

ESM 1 (DOCX 79.2 KB)

## Appendix

**Table 6:** Description of the data setup based on one case

ID	Observation	Year	Quarter	Employment	Tenure	Age	Firm ID	Mass Layoff	Included Into Our Sample	Pregnancy
1	1	1981	1	Other	––	24	––	––	Not included	No
1	2	1981	2	Work	0.25	24	12	No	Not included	No
1	3	1981	3	Work	0.50	24	12	No	Not included	No
1	4	1981	4	Work	0.75	24	12	No	Not included	No
1	5	1982	1	Work	1.00	25	12	No	Not included	No
1	6	1982	2	Work	1.25	25	12	No	Not included	No
1	7	1982	3	Work	1.50	25	12	No	Not included	No
1	8	1982	4	Work	1.75	25	12	No	Control	No
1	9	1983	1	Work	2.00	26	12	No	Control	No
1	10	1983	2	Work	2.25	26	12	No	Control	No
1	11	1983	3	Work	2.50	26	12	Yes	Control	No
1	12	1983	4	Work	2.75	26	12	Yes	Control	No
1	13	1984	1	Work	3.00	27	12	Yes	Treated	No
1	14	1984	2	Other	––	27	––	Yes	Not included	No
1	15	1984	3	Other	––	27	––	––	Not included	No
1	16	1984	4	Other	––	27	––	––	Not included	No
1	17	1985	1	Other	––	28	––	––	Not included	Yes

*Notes:* **Entry into our analytical sample**: ID 1 enters the data set in the first quarter of 1981. She starts working in the second quarter of 1981. She will have acquired 1.5 years of tenure at the beginning of the fourth quarter of 1982, when she enters our analytical sample. **Exit from our analytical sample:** After entering our analytical sample, the woman is working for six quarters and then stops working. Because ID 1 exits the labor market, she leaves our sample. The units of observation are quarters. Thus, ID 1 enters our data set with six observations. **Distinction between the treatment and the control group:** From the firm identifier, we know that there was a mass layoff in the firm between July 1982 and June 1983 (we only have information on this characteristic of the firm every June of a given year). We assume that the exit from the labor market is due to the mass layoff. Therefore, observation number 13 of this woman enters our analytical sample as a treatment observation. Observations 8 to 12 enter our analytical sample as control observations. **Linking employment and fertility**: For each single observation, we calculate the cumulated pregnancy probability by the end of years 1, 2, 3, 4, and 5 after the displacement (for the treated cases), and after the “reference quarter” for the control group. These additional five variables are not displayed.

## Footnotes

1 The public pension fund regularly verifies employment and fertility histories through letters sent to the insured over age 30 (“clearance” of the pension records). BASiD contains a variable on the most recent clearance date. For more details about clearance in relation to fertility histories, see Kreyenfeld and Mika (2008). Women whose last clearance was before they reached age 30 are most likely a selective group. For example, these women may have successfully applied for a disability pension. The incidence of these cases in the BASiD data increases after 2003.

2 In our sample, approximately 4 % of the treated observations are repeated treatments. Keeping quarterly observations of treated women only until the first treatment (i.e., focusing on first treatments only) has no impact on our results.

3 Alternatively, we could exclude from the sample the women who left employment in the absence of a mass layoff or a plant closure. We include them in the control group because these women might have entered non-employment because they were planning to have a child or had become pregnant. However, our results are robust if we exclude this group from the control group.

4 We cannot include individual fixed effects because we are investigating first births. Thus, no woman in our sample became pregnant before quarter *q* + 1.

5 Our results are robust to using the regional unemployment rate on the state level (available upon request).

6 We apply IPW estimators as outlined earlier, but we use a time-varying employment indicator (1 if employed, and 0 otherwise) as an outcome. The results are very similar when we estimate LPMs.

7 We calculate the relative effects using the outcome values of the control group (7.2 % for year 1, 18.4 % for year 3, and 25.7 % for year 5) as baseline.

8 For the LPM, the coefficients are significantly different at the 5 % level. For the IPW estimates, the difference for the birth rate after three years is statistically significant at the 5 % level, and the difference after four years is significant at the 10 % level (results available upon request).

9 Del Bono et al. (2012) did not report the results for first birth in their article. However, they made their data publicly available, which allowed us to reestimate the model for first births.
