# Supplementary material for: Job Displacement and First Birth Over the Business Cycle
Source: Demography. 2017 Jun 5;54(3):933–59. doi: 10.1007/s13524-017-0580-4 (PMC5486876; doi:10.1007/s13524-017-0580-4)
Supplement: Supplementary file 1 — (DOCX 79.2 KB) [file 13524_2017_580_MOESM1_ESM.docx]

**Online Resource 1**

**Job Displacement and First Birth Over the Business Cycle**

Barbara Hofmann, Michaela Kreyenfeld, and Arne Uhlendorff

| Table S1: Descriptive statistics. Full set of control variables | | | | | | | | | | | | | |  |
| --- | --- | --- | --- | --- | --- | --- | --- | --- | --- | --- | --- | --- | --- | --- |
|  | Downturn |  |  |  |  | No downturn | |  | |  | |  | |  |
|  | Treated | Control^a)^ | | |  | Treated | Control^a)^ | | | | | |  | |
|  |  | Unweighted |  | Weighted |  |  | Unweighted | |  | | Weighted | |  | |
| *Individual characteristics* |  |  |  |  |  |  |  | |  | |  | |  | |
| Tenure > 2.5 years | 0.672 | 0.790 | *** | 0.671 |  | 0.714 | 0.814 | | *** | | 0.716 | |  | |
| Wage quarter -1 | 49.493 | 53.314 | *** | 49.439 |  | 47.867 | 52.663 | | *** | | 47.810 | |  | |
| -2 | 49.023 | 53.040 | *** | 48.975 |  | 47.938 | 52.207 | | *** | | 47.929 | |  | |
| -3 | 48.715 | 52.736 | *** | 48.666 |  | 47.954 | 51.725 | | *** | | 47.960 | |  | |
| -4 | 48.010 | 52.451 | *** | 47.964 |  | 46.787 | 51.248 | | *** | | 46.754 | |  | |
| -5 | 47.737 | 51.966 | *** | 47.689 |  | 46.612 | 50.730 | | *** | | 46.602 | |  | |
| -6 | 46.834 | 50.999 | *** | 46.778 |  | 45.804 | 49.764 | | *** | | 45.772 | |  | |
| -7 | 45.501 | 49.504 | *** | 45.445 |  | 43.108 | 48.418 | | *** | | 43.275 | |  | |
| -8 | 43.711 | 48.145 | *** | 43.658 |  | 42.406 | 47.177 | | *** | | 42.416 | |  | |
| -9 | 41.288 | 46.761 | *** | 41.209 |  | 41.065 | 46.004 | | *** | | 41.058 | |  | |
| -10 | 40.249 | 45.447 | *** | 40.168 |  | 40.957 | 44.882 | | *** | | 40.952 | |  | |
| -11 | 39.335 | 44.250 | *** | 39.254 |  | 39.708 | 43.858 | | *** | | 39.722 | |  | |
| -12 | 38.093 | 43.100 | *** | 37.978 |  | 39.181 | 42.872 | | *** | | 39.193 | |  | |
| Employed quarter q-7 | 0.971 | 0.982 | * | 0.971 |  | 0.978 | 0.983 | |  | | 0.980 | |  | |
| -8 | 0.937 | 0.966 | *** | 0.937 |  | 0.951 | 0.969 | | ** | | 0.950 | |  | |
| -9 | 0.915 | 0.951 | *** | 0.916 |  | 0.930 | 0.955 | | ** | | 0.930 | |  | |
| -10 | 0.888 | 0.937 | *** | 0.888 |  | 0.927 | 0.943 | | † | | 0.927 | |  | |
| -11 | 0.880 | 0.924 | *** | 0.880 |  | 0.913 | 0.932 | | † | | 0.913 | |  | |
| -12 | 0.876 | 0.912 | ** | 0.876 |  | 0.904 | 0.922 | |  | | 0.904 | |  | |
| Age 25-27 | 0.291 | 0.312 |  | 0.292 |  | 0.294 | 0.316 | |  | | 0.296 | |  | |
| Age 28-30 | 0.263 | 0.237 |  | 0.264 |  | 0.221 | 0.231 | |  | | 0.223 | |  | |
| Age 31-33 | 0.198 | 0.174 |  | 0.199 |  | 0.202 | 0.168 | | * | | 0.200 | |  | |
| Age 34-36 | 0.106 | 0.136 | * | 0.106 |  | 0.130 | 0.134 | |  | | 0.131 | |  | |
| Age 37-38 | 0.080 | 0.078 |  | 0.078 |  | 0.087 | 0.078 | |  | | 0.084 | |  | |
| Age 39-40 | 0.062 | 0.064 |  | 0.062 |  | 0.066 | 0.072 | |  | | 0.067 | |  | |
| Agricultural, low skilled manual | 0.143 | 0.120 | † | 0.140 |  | 0.124 | 0.122 | |  | | 0.124 | |  | |
| Skilled manual, technician, engineer | 0.106 | 0.110 |  | 0.106 |  | 0.125 | 0.115 | |  | | 0.121 | |  | |
| Low, medium skilled services | 0.167 | 0.142 | † | 0.168 |  | 0.179 | 0.138 | | ** | | 0.181 | |  | |
| Semi-/ professional | 0.057 | 0.114 | *** | 0.057 |  | 0.057 | 0.117 | | *** | | 0.058 | |  | |
| Low skilled commercial, administration | 0.125 | 0.125 |  | 0.125 |  | 0.151 | 0.131 | |  | | 0.153 | |  | |
| Skilled commercial, administration | 0.400 | 0.388 |  | 0.400 |  | 0.363 | 0.376 | |  | | 0.364 | |  | |
| *Firm variables* |  |  |  |  |  |  |  | |  | |  | |  | |
| Sector energy, water, mining | 0.033 | 0.026 |  | 0.032 |  | 0.022 | 0.029 | |  | | 0.022 | |  | |
| Manufacturing industry | 0.280 | 0.290 |  | 0.280 |  | 0.287 | 0.289 | |  | | 0.286 | |  | |
| Retail, wholesale | 0.207 | 0.146 | *** | 0.207 |  | 0.232 | 0.153 | | *** | | 0.233 | |  | |
| Transportation, telecommunication | 0.041 | 0.028 | † | 0.041 |  | 0.048 | 0.026 | | *** | | 0.046 | |  | |
| Financial sector, insurances | 0.059 | 0.086 | * | 0.057 |  | 0.054 | 0.081 | | * | | 0.054 | |  | |
| Services | 0.325 | 0.313 |  | 0.326 |  | 0.314 | 0.311 | |  | | 0.315 | |  | |
| Non-profit sector | 0.010 | 0.027 | ** | 0.010 |  | 0.011 | 0.025 | | * | | 0.011 | |  | |
| Wage 25-% tile | 39.504 | 43.035 | *** | 39.445 |  | 41.195 | 43.332 | | ** | | 41.135 | |  | |
| Wage median | 51.517 | 56.191 | *** | 51.462 |  | 53.638 | 56.846 | | *** | | 53.600 | |  | |
| Wage 75-% tile | 63.989 | 70.180 | *** | 63.941 |  | 67.379 | 71.186 | | *** | | 67.377 | |  | |
| Share of employees < age 30 | 0.350 | 0.342 |  | 0.350 |  | 0.354 | 0.340 | | * | | 0.354 | |  | |
| Share of employees ≥ age 30 & < age 50 | 0.487 | 0.479 |  | 0.486 |  | 0.484 | 0.484 | |  | | 0.484 | |  | |
| Share of employees ≥ age 50 | 0.163 | 0.179 | *** | 0.163 |  | 0.162 | 0.176 | | ** | | 0.163 | |  | |
| Share of female workers | 0.582 | 0.575 |  | 0.583 |  | 0.580 | 0.574 | |  | | 0.581 | |  | |
| Share low qualified | 0.211 | 0.226 | † | 0.211 |  | 0.215 | 0.230 | | † | | 0.215 | |  | |
| Firm size 4-10 employees | 0.237 | 0.137 | *** | 0.238 |  | 0.243 | 0.132 | | *** | | 0.242 | |  | |
| Firm size 11-50 | 0.259 | 0.194 | *** | 0.259 |  | 0.255 | 0.199 | | *** | | 0.256 | |  | |
| Firm size 51-250 | 0.249 | 0.247 |  | 0.249 |  | 0.238 | 0.254 | |  | | 0.237 | |  | |
| Firm size 251-1999 | 0.255 | 0.423 | *** | 0.253 |  | 0.264 | 0.415 | | *** | | 0.265 | |  | |
| Notes: Table displays means of control variables separately for the treated and controls by downturn and upturn. For the control group unweighted and weighted means are presented. The weights (single weighting) are explained in the text. a) Significance of differences (t-test): †: 10 %, *: 5 %, **: 1 %, ***: 0.1%. | | | | | | | | | | | | | |  |

**Robustness Checks**

We have conducted several robustness checks not reported in the main paper. First, to check how sensitive our results are to the decision to use a binary indicator of an economic downturn, we re-estimated one of the main specifications (Table 4, Model C), interacting the treatment dummy with the cyclical component of the unemployment rate. The results are reported in Table S2 (Model A1). We also conducted this check for the models that included the parental leave reform interaction terms (Table 5, Models G and H). The results from this robustness check are also available in Table S2 (Model A2 and A3). The results show that our main finding is qualitatively robust: the higher the cyclical component, the stronger the effect of displacement on fertility; i.e., the level of the unemployment rate is relative to the predicted trend.

Second, we investigate whether using the GDP as the indicator of the business cycle changes our results qualitatively, and we find that our results are robust (Table S3). The findings indicate that our results do not depend on whether we use the unemployment rate or the GDP as an indicator of the business cycle.

Finally, we have restricted our control group to women who were not laid off due to a mass layoff or a firm closure in the upcoming four quarters. This ensures that we do not have a strong overlap between the treatment and the control groups. The results based on the LPM are not affected by this restriction (Table S4).

| Table S2: Robustness checks with the cyclical component of the unemployment rate | | | | | | |  |
| --- | --- | --- | --- | --- | --- | --- | --- |
| Year after layoff | Model A1: LPM | | Model A2: LPM with continuous unemployment rate and reform effects (1986) | | Model A3: LPM with continuous unemployment rate and reform effects (1992) | |  |
|  | Treated | Treated × cyclical component of the unemployment rate | Treated | Treated × cyclical unemployment-rate | Treated | Treated × cyclical unemployment-rate |  |
| 1 | -0.017** | -0.004 | 0.002 | -0.005 | -0.003 | -0.004 |  |
|  | (0.006) | (0.006) | (0.015) | (0.006) | (0.012) | (0.006) |  |
| 2 | -0.015 | -0.015† | -0.008 | -0.016† | -0.014 | -0.015† |  |
|  | (0.009) | (0.008) | (0.018) | (0.008) | (0.015) | (0.008) |  |
| 3 | -0.013 | -0.024* | -0.022 | -0.023* | -0.014 | -0.024* |  |
|  | (0.011) | (0.010) | (0.020) | (0.010) | (0.017) | (0.010) |  |
| 4 | -0.009 | -0.029** | -0.010 | -0.029** | -0.004 | -0.028* |  |
|  | (0.013) | (0.011) | (0.022) | (0.011) | (0.018) | (0.011) |  |
| 5 | -0.013 | -0.026* | -0.018 | -0.026* | -0.013 | -0.025* |  |
|  | (0.014) | (0.011) | (0.023) | (0.012) | (0.019) | (0.012) |  |
| Model A1: Interacting of the treatment dummy with the cyclical component of the unemployment rate  Model A2: Same as Model A1 including interaction effects for 1986 parental leave reform  Model A3: Same as Model A1 including interaction effects for 1992 parental leave reform  Significance levels: †: 10 %, *: 5 %, **: 1 %, ***: 0.1%. | | | | | | | |

| Table S3: Robustness checks based on gross domestic product | | | | | | | | | | | |
| --- | --- | --- | --- | --- | --- | --- | --- | --- | --- | --- | --- |
| Year after layoff | Model A4: LPM | | Model A5: IPW | | Model A6: Double IPW | | Model A7: LPM with reform effects (1992) | | Model A8: LPM with continuous GDP-rate and reform effects (1992) | |  |
|  | Treated in downturn | Treated in upturn | Treated in downturn | Treated in upturn | Treated in downturn | Treated | Treated in downturn | Treated | Treated | Treated × cyclical GDP-rate |  |
|  |  |  |  |  |  | in |  | in |  |  |  |
|  |  |  |  |  |  | upturn |  | upturn |  |  |  |
| 1 | -0.021* | -0.014 | -0.021* | -0.013 | -0.021* | -0.022* | -0.008 | 0.004 | -0.003 | 0.015 |  |
|  | (0.010) | (0.009) | (0.010) | (0.009) | (0.010) | (0.009) | (0.012) | (0.016) | (0.012) | (0.025) |  |
| 2 | -0.034** | 0.004 | -0.034* | 0.005 | -0.034* | -0.003 | -0.030† | 0.010 | -0.013 | 0.055 |  |
|  | (0.013) | (0.013) | (0.013) | (0.014) | (0.013) | (0.016) | (0.016) | (0.020) | (0.015) | (0.035) |  |
| 3 | -0.036* | 0.008 | -0.035* | 0.014 | -0.035* | 0.009 | -0.032† | 0.013 | -0.013 | 0.078† |  |
|  | (0.015) | (0.017) | (0.016) | (0.017) | (0.016) | (0.021) | (0.019) | (0.022) | (0.017) | (0.041) |  |
| 4 | -0.035* | 0.014 | -0.033* | 0.030 | -0.033† | 0.012 | -0.023 | 0.025 | -0.003 | 0.098† |  |
|  | (0.016) | (0.020) | (0.017) | (0.020) | (0.017) | (0.026) | (0.020) | (0.024) | (0.018) | (0.046) |  |
| 5 | -0.035* | 0.011 | -0.034† | 0.011 | -0.035† | -0.011 | -0.031 | 0.014 | -0.012 | 0.077 |  |
|  | (0.017) | (0.022) | (0.018) | (0.022) | (0.019) | (0.030) | (0.021) | (0.025) | (0.019) | (0.048) |  |
| Notes: Dependent variable: cumulated first birth probability.  Model A4: Linear probability model for pooled sample including interaction terms  Model A5: Inverse probability weighting (IPW) estimation for separate samples (upturn/downturn)  Model A6: Double IPW estimation for separate samples (upturn/downturn). For IPW estimators standard errors are bootstrapped (500 replications).  Model A7: Accounting for changes in the parental leave system in 1992. In this model, we allow a shift of the treatment effect after the reform, independent of the status of the business cycle.  Model A8: As in Model A7, we allow a shift of the treatment effect after the reform 1992, independent of the status of the business cycle. We interact the treatment indicator with the standardized cyclical component of the yearly GDP.  Significance levels: †: 10 %, *: 5 %, **: 1 %, ***: 0.1%. | | | | | | | | | | |  |

| Table S4: Robustness check. Effect of a layoff by business cycle based on a restricted control group | | |
| --- | --- | --- |
| Year after layoff | Model A9: LPM | |
|  | Treated  in  downturn | Treated  in  upturn |
| 1 | -0.021* | -0.015 |
|  | (0.009) | (0.010) |
| 2 | -0.028* | 0.001 |
|  | (0.013) | (0.015) |
| 3 | -0.034* | 0.015 |
|  | (0.014) | (0.019) |
| 4 | -0.032* | 0.023 |
|  | (0.016) | (0.023) |
| 5 | -0.032† | 0.011 |
|  | (0.018) | (0.023) |
| Notes: Dependent variable: cumulated first birth probability.  Significance levels: †: 10 %, *: 5 %, **: 1 %, ***: 0.1%. | | |

| Table S5: Logit models for constructing the weights for IPW estimations | | | | | | | | | | |
| --- | --- | --- | --- | --- | --- | --- | --- | --- | --- | --- |
|  | Logit A | | Logit B | | Logit C | | Logit D | | | |
|  | Coeff. | (st.err.) | Coeff. | (st.err.) | Coeff. | (st.err.) | Coeff. | (st.err.) | |  |
| Tenure > 2.5 years | -0.507*** | (0.076) | -0.525*** | (0.108) | -0.479*** | (0.108) | 0.149 | (0.158) | |  |
| Wage quarter -1 | -0.005 | (0.004) | -0.013* | (0.005) | 0.004 | (0.006) | -0.019† | (0.010) | |  |
| -2 | -0.001 | (0.005) | 0.002 | (0.007) | -0.004 | (0.007) | 0.011 | (0.011) | |  |
| -3 | 0.005 | (0.005) | 0.008 | (0.006) | 0.001 | (0.007) | 0.005 | (0.008) | |  |
| -4 | -0.003 | (0.005) | -0.004 | (0.008) | -0.002 | (0.007) | -0.015 | (0.016) | |  |
| -5 | -0.002 | (0.006) | -0.002 | (0.008) | -0.003 | (0.008) | 0.013 | (0.017) | |  |
| -6 | 0.003 | (0.004) | 0.009 | (0.006) | -0.002 | (0.005) | 0.005 | (0.008) | |  |
| -7 | -0.005 | (0.003) | -0.013** | (0.004) | 0.003 | (0.005) | -0.011* | (0.006) | |  |
| -8 | 0.009* | (0.003) | 0.007 | (0.005) | 0.009* | (0.005) | -0.010 | (0.007) | |  |
| -9 | -0.008* | (0.004) | -0.007 | (0.005) | -0.008† | (0.005) | -0.003 | (0.008) | |  |
| -10 | 0.005 | (0.004) | 0.008 | (0.005) | 0.002 | (0.005) | 0.010 | (0.009) | |  |
| -11 | -0.002 | (0.003) | -0.006 | (0.005) | 0.001 | (0.005) | -0.003 | (0.007) | |  |
| -12 | 0.002 | (0.003) | 0.005 | (0.004) | -0.002 | (0.004) | 0.005 | (0.005) | |  |
| Employed quarter q-7 | 0.222 | (0.234) | 0.508 | (0.344) | -0.015 | (0.322) | 0.364 | (0.505) | |  |
| -8 | -0.165 | (0.219) | 0.015 | (0.315) | -0.318 | (0.308) | 0.432 | (0.468) | |  |
| -9 | -0.197 | (0.214) | -0.491 | (0.313) | 0.028 | (0.294) | -0.425 | (0.439) | |  |
| -10 | 0.038 | (0.219) | 0.356 | (0.327) | -0.228 | (0.297) | 0.511 | (0.486) | |  |
| -11 | 0.105 | (0.211) | 0.178 | (0.303) | 0.024 | (0.295) | -0.014 | (0.466) | |  |
| -12 | -0.015 | (0.169) | -0.108 | (0.243) | 0.111 | (0.234) | -0.051 | (0.342) | |  |
| Age 25-27 | -0.108 | (0.129) | -0.125 | (0.177) | -0.100 | (0.188) | -0.055 | (0.266) | |  |
| Age 28-30 | 0.041 | (0.129) | -0.027 | (0.179) | 0.105 | (0.186) | -0.266 | (0.267) | |  |
| Age 31-33 | 0.182 | (0.130) | 0.250 | (0.179) | 0.112 | (0.189) | -0.023 | (0.269) | |  |
| Age 34-36 | -0.082 | (0.140) | 0.025 | (0.190) | -0.220 | (0.207) | 0.223 | (0.291) | |  |
| Age 37-38 | 0.101 | (0.151) | 0.139 | (0.207) | 0.035 | (0.220) | -0.019 | (0.312) | |  |
| Medium/high skill manual/techn. | -0.225† | (0.126) | -0.052 | (0.176) | -0.417* | (0.180) | 0.395 | (0.255) | |  |
| Service | -0.207 | (0.126) | 0.015 | (0.178) | -0.440* | (0.180) | 0.418 | (0.257) | |  |
| Professional | -0.719*** | (0.169) | -0.569* | (0.239) | -0.867*** | (0.239) | 0.351 | (0.348) | |  |
| Low skilled office/admin. | -0.184 | (0.126) | 0.001 | (0.176) | -0.373* | (0.180) | 0.441† | (0.252) | |  |
| High skilled off./adm./managem. | -0.202† | (0.115) | -0.092 | (0.164) | -0.307† | (0.160) | 0.184 | (0.236) | |  |
| Sector energy, water, mining | 1.029*** | (0.234) | 0.966** | (0.361) | 1.136*** | (0.310) | 0.047 | (0.476) | |  |
| Manufacturing industry | 0.633*** | (0.163) | 0.942*** | (0.246) | 0.358 | (0.219) | 0.521 | (0.330) | |  |
| Retail, wholesale | 0.792*** | (0.159) | 1.087*** | (0.240) | 0.509* | (0.214) | 0.551† | (0.328) | |  |
| Transportation and telecomm. | 0.926*** | (0.202) | 1.321*** | (0.295) | 0.560* | (0.282) | 0.646 | (0.412) | |  |
| Financial sector, insurances | 0.361† | (0.190) | 0.542† | (0.283) | 0.215 | (0.259) | 0.389 | (0.393) | |  |
| Services | 0.614*** | (0.158) | 0.835*** | (0.240) | 0.420* | (0.211) | 0.451 | (0.325) | |  |
| Non-profit sector | -0.212 | (0.316) | 0.081 | (0.445) | -0.497 | (0.456) | 0.612 | (0.659) | |  |
| Wage 25-% tile | 0.005 | (0.004) | 0.012* | (0.006) | -0.003 | (0.006) | 0.008 | (0.008) | |  |
| Wage median | -0.001 | (0.005) | -0.007 | (0.007) | 0.006 | (0.008) | -0.009 | (0.011) | |  |
| Wage 75-% tile | -0.006† | (0.003) | 0.002 | (0.005) | -0.014** | (0.005) | 0.021** | (0.007) | |  |
| Share of employees < age 30 | -0.378† | (0.212) | -0.059 | (0.296) | -0.736* | (0.307) | 0.639 | (0.407) | |  |
| Share of employees ≥ age 50 | -0.482 | (0.309) | 0.016 | (0.436) | -1.007* | (0.444) | 0.055 | (0.601) | |  |
| Share of female workers | -0.256† | (0.148) | -0.036 | (0.210) | -0.476* | (0.208) | 0.288 | (0.300) | |  |
| Share low qualified | -0.102 | (0.169) | 0.014 | (0.240) | -0.212 | (0.240) | 0.462 | (0.323) | |  |
| Caldendar year | -0.034† | (0.018) | -0.065 | (0.054) | -0.039† | (0.022) | - |  | |  |
| Caldendar year squared | 0.002** | (0.001) | 0.003† | (0.002) | 0.002** | (0.001) | - |  | |  |
| Quarter1 (Jan.-March) | -1.508*** | (0.092) | -1.592*** | (0.132) | -1.419*** | (0.129) | -0.110 | (0.193) | |  |
| Quarter2 (May-July) | -1.171*** | (0.082) | -1.251*** | (0.117) | -1.096*** | (0.115) | -0.147 | (0.171) | |  |
| Quarter3 (Aug.-Oct.) | -0.961*** | (0.075) | -0.940*** | (0.103) | -0.993*** | (0.109) | 0.138 | (0.155) | |  |
| Firm size 4-10 employees | -0.274** | (0.086) | -0.347** | (0.121) | -0.208† | (0.124) | 0.015 | (0.178) | |  |
| Firm size 11-50 | -0.552*** | (0.095) | -0.650*** | (0.133) | -0.460*** | (0.136) | -0.124 | (0.193) | |  |
| Firm size 51-250 | -0.927*** | (0.101) | -0.975*** | (0.140) | -0.887*** | (0.148) | -0.062 | (0.211) | |  |
| Constant | -2.549*** | (0.395) | -3.863*** | (0.637) | -1.171* | (0.532) | -2.710*** | (0.772) | |  |
| Number of observations | 115,058 |  | 60,709 |  | 54,349 |  | 1,257 |  | |  |
| Notes: Model Logit A: Logistic regression for the probability of being laid off based on the full sample. Corresponding weights are used in Model B, Table 3.  Model Logit B: Logistic regression for the probability of being laid off based on periods with high unemployment rates. Corresponding weights are used in Model D (first column), Table 4.  Model Logit C: Logistic regression for the probability of being laid off based on periods with low unemployment rates. Corresponding weights are used in Model D (second column), Table 4.  Model Logit D: Logistic regression for the probability of being laid off in periods with low unemployment rates based on the sample of all women who have been displaced due to a mass layoff or a plant closure. Corresponding weights are used in Model E, Table 4.  Significance levels: †: 10 %, *: 5 %, **: 1 %, ***: 0.1%. | | | | | | | | | | |
| Table S6: Linear probability models for the impact of a layoff on fertility in the first year after layoff   \|  \| Model A \| \| Model C \| \| \| --- \| --- \| --- \| --- \| --- \| \|  \| Coeff. \| (st. err.) \| Coeff. \| (st. err.) \| \| Treated \| -0.017** \| (0.006) \|  \|  \| \| Treated × downturn \|  \|  \| -0.020* \| (0.009) \| \| Treated × no downturn \|  \|  \| -0.014 \| (0.009) \| \| Downturn \|  \|  \| 0.002 \| (0.003) \| \| Tenure > 2.5 years \| -0.008† \| (0.004) \| -0.008† \| (0.004) \| \| Wage quantile in quarter -1 \| -0.007 \| (0.010) \| -0.007 \| (0.010) \| \| -2 \| -0.004 \| (0.008) \| -0.004 \| (0.008) \| \| -3 \| 0.011 \| (0.008) \| 0.011 \| (0.008) \| \| -4 \| -0.000 \| (0.009) \| -0.000 \| (0.009) \| \| -5 \| 0.003 \| (0.008) \| 0.003 \| (0.008) \| \| -6 \| 0.004 \| (0.007) \| 0.004 \| (0.007) \| \| -7 \| -0.001 \| (0.006) \| -0.001 \| (0.006) \| \| -8 \| 0.004 \| (0.006) \| 0.004 \| (0.006) \| \| -9 \| 0.008 \| (0.006) \| 0.008 \| (0.006) \| \| -10 \| 0.003 \| (0.006) \| 0.003 \| (0.006) \| \| -11 \| 0.000 \| (0.000) \| 0.000 \| (0.000) \| \| -12 \| 0.000 \| (0.000) \| 0.000 \| (0.000) \| \| Share employed in quarter -7 \| -0.003 \| (0.005) \| -0.003 \| (0.005) \| \| -8 \| 0.008† \| (0.005) \| 0.008† \| (0.005) \| \| -9 \| 0.001 \| (0.004) \| 0.001 \| (0.004) \| \| -10 \| -0.000 \| (0.004) \| -0.000 \| (0.004) \| \| -11 \| 0.008† \| (0.005) \| 0.008† \| (0.005) \| \| -12 \| 0.007 \| (0.005) \| 0.007 \| (0.005) \| \| Age 25-27 \| 0.096*** \| (0.004) \| 0.096*** \| (0.004) \| \| Age 28-30 \| 0.091*** \| (0.004) \| 0.091*** \| (0.004) \| \| Age 31-33 \| 0.058*** \| (0.004) \| 0.058*** \| (0.004) \| \| Age 34-36 \| 0.029*** \| (0.003) \| 0.029*** \| (0.003) \| \| Age 37-38 \| 0.007** \| (0.003) \| 0.007** \| (0.003) \| \| Medium high sk. manual/tech. \| -0.007 \| (0.007) \| -0.007 \| (0.007) \| \| Service \| -0.007 \| (0.007) \| -0.008 \| (0.007) \| \| Professional \| 0.004 \| (0.008) \| 0.004 \| (0.008) \| \| Low skilled office/admin. \| -0.009 \| (0.007) \| -0.009 \| (0.007) \| \| High skilled off./adm./managem. \| -0.013* \| (0.006) \| -0.013* \| (0.006) \| \| Sector energy, water, mining \| -0.005 \| (0.010) \| -0.005 \| (0.010) \| \| Manufacturing industry \| -0.003 \| (0.006) \| -0.003 \| (0.006) \| \| Retail, wholesale \| -0.006 \| (0.006) \| -0.006 \| (0.006) \| \| Transportation and telecomm. \| -0.006 \| (0.010) \| -0.006 \| (0.010) \| \| Financial sector, insurances \| -0.001 \| (0.007) \| -0.001 \| (0.007) \| \| Services \| -0.002 \| (0.006) \| -0.002 \| (0.006) \| \| Non-profit sector \| -0.007 \| (0.010) \| -0.007 \| (0.010) \| \| Wage 25-% tile \| 0.004 \| (0.022) \| 0.004 \| (0.022) \| \| Wage median \| -0.022 \| (0.029) \| -0.022 \| (0.029) \| \| Wage 75-% tile \| 0.005 \| (0.019) \| 0.005 \| (0.019) \| \| Share of employees < age 30 \| 0.021† \| (0.012) \| 0.022† \| (0.012) \| \| Share of employees ≥ age 50 \| -0.044** \| (0.017) \| -0.043** \| (0.017) \| \| Share of female workers \| -0.005 \| (0.008) \| -0.005 \| (0.008) \| \| Share low qualified \| -0.001 \| (0.009) \| -0.001 \| (0.009) \| \| Time trend \| 0.002* \| (0.001) \| 0.002† \| (0.001) \| \| Time squared \| -0.000† \| (0.000) \| -0.000 \| (0.000) \| \| Quarter1 (Jan.-March) \| 0.005*** \| (0.001) \| 0.005*** \| (0.001) \| \| Quarter2 (May-July) \| 0.004*** \| (0.001) \| 0.004*** \| (0.001) \| \| Quarter3 (Aug.-Oct.) \| 0.001* \| (0.001) \| 0.001* \| (0.001) \| \| Firm size 4-10 employees \| 0.000 \| (0.005) \| 0.000 \| (0.005) \| \| Firm size 11-50 \| -0.002 \| (0.005) \| -0.001 \| (0.005) \| \| Firm size 51-250 \| -0.003 \| (0.005) \| -0.003 \| (0.005) \| \| Constant \| -0.010 \| (0.018) \| -0.010 \| (0.018) \| \| Number of observations \| 115,058 \|  \| 115,058 \|  \| \| Notes: Dependent variable: Cumulated first birth probability after one year. The table displays the full list of coefficients of Model A (Table 3) and Model C (Table 4) of the main paper. Significance levels: †: 10 %, *: 5 %, **: 1 %, ***: 0.1%. \| \| \| \| \| | | | | | | | | | |  |
